# Efficacy and neural mechanisms of transcutaneous auricular vagus nerve stimulation for chronic insomnia: a study protocol for a randomized controlled trial

**DOI:** 10.3389/fpsyt.2026.1944503

**Published:** 2026-09-28

**Authors:** Menghan Feng, Qin Liu, Xin Chen, Wenting Luo, Mengxi Zhang, Weifeng Zhu

**Affiliations:** 1 The Affiliated Guangzhou Hospital of TCM of Guangzhou University of Chinese Medicine, Guangzhou, China; 2 The Affiliated Traditional Chinese Medicine Hospital, Guangzhou Medical University, Guangzhou, China; 3 Sleep Research Institute of Traditional Chinese Medicine, Guangzhou Medical University, Guangzhou, China

**Keywords:** chronic insomnia disorder, functional magnetic resonance imaging, neuromodulation, randomized controlled trial, study protocol, transcutaneous auricular vagus nerve stimulation

## Abstract

**Background:**

Traditional auricular therapy for chronic insomnia (*Bu Mei* in TCM) is used in clinical practice but is limited by insufficient standardization and poorly understood mechanisms. Transcutaneous auricular vagus nerve stimulation (taVNS) provides a quantifiable and reproducible neuromodulatory approach by stimulating vagally innervated regions of the auricular concha, which overlap with auricular acupoints used in TCM. However, evidence in insomnia remains predominantly based on clinical outcomes, whereas longitudinal neuroimaging evidence is limited. Resting-state functional magnetic resonance imaging (rs-fMRI) may complement clinical assessments by characterizing spontaneous brain activity and treatment-related functional changes, thereby providing objective neuroimaging evidence relevant to the potential mechanisms of taVNS.

**Methods:**

This protocol will recruit 60 patients with chronic insomnia disorder (CID) and 30 age- and sex-matched healthy controls (HCs). Patients with CID will be randomized 1:1 to receive 4 weeks of active or sham-taVNS. Resting-state functional magnetic resonance imaging (rs-fMRI) will be performed at baseline in all participants and repeated at Week 4 in patients with CID, within 24 hours after the final intervention session. Fractional amplitude of low-frequency fluctuation (fALFF) will serve as a prespecified exploratory neuroimaging measure, with amplitude of low-frequency fluctuation (ALFF) included as a complementary exploratory measure. The neuroimaging analyses will examine baseline differences between patients with CID and HCs and between-group differences in baseline-to-Week-4 changes between the active and sham-taVNS groups to characterize brain functional alterations associated with CID and changes associated with active taVNS relative to sham-taVNS. The primary clinical outcome will be the change in Pittsburgh Sleep Quality Index (PSQI) score from baseline to Week 4. Secondary and follow-up assessments will evaluate insomnia severity, daytime sleepiness, emotional status, cognitive function, pre-sleep arousal, and quality of life. Exploratory correlations between changes in neuroimaging measures and clinical outcomes will also be examined.

**Discussion:**

This study will integrate clinical assessments with rs-fMRI to evaluate the clinical effects of taVNS in patients with CID and explore associated changes in spontaneous brain activity. The integration of clinical outcomes and exploratory neuroimaging measures may provide complementary evidence on treatment effects and associated brain functional changes, thereby informing future investigation of the potential neural mechanisms of taVNS in CID.

**Clinical Trial Registration:**

https://www.chictr.org.cn/hvshowprojectEN.html?id=277442&v=1.0, identifier ChiCTR2500103157.

## Introduction

Chronic insomnia disorder (CID) is a prevalent sleep–wake disorder characterized by persistent difficulty initiating sleep, maintaining sleep, or early-morning awakening, occurring at least three nights per week for at least three months and accompanied by daytime functional impairment, including fatigue, impaired attention, memory complaints, mood disturbance, or reduced quality of life. Diagnosis requires that insomnia symptoms persist despite adequate opportunity and circumstances for sleep, cause daytime impairment, and are not better explained by another sleep–wake disorder or other relevant factors (1). Epidemiological evidence suggests that approximately 10% of adults meet the diagnostic criteria for chronic insomnia disorder, whereas a larger proportion experience intermittent or subthreshold insomnia symptoms. Women, older adults, and individuals with lower socioeconomic status or comorbid medical and psychiatric conditions are at increased risk (2).

Current treatment strategies for CID mainly include behavioral, psychological, pharmacological, and physical interventions. Cognitive behavioral therapy for insomnia (CBT-I) is recommended as a first-line treatment in major clinical guidelines; however, its implementation is often limited by the insufficient availability of trained therapists, uneven access to specialized sleep services, treatment adherence barriers, and relatively high time and economic costs (1, 3). Pharmacological treatments may provide short-term symptomatic relief. Nevertheless, concerns remain regarding residual daytime sedation, tolerance, dependence, inappropriate long-term use, and other adverse effects, particularly with benzodiazepines and Z-drugs (4). Therefore, safe, standardized, portable, and home-applicable non-pharmacological interventions for CID warrant further investigation.

From the perspective of traditional Chinese medicine (TCM), CID corresponds to the concept of *Bu Mei* (sleeplessness). TCM-based non-pharmacological interventions, particularly auricular acupoint stimulation, have been widely used for insomnia management. According to standard TCM auricular microsystem mapping, the *Liver* and *Kidney* acupoints, which are traditionally associated with sleep regulation, are located within the cymba conchae. Neuroanatomical studies have demonstrated substantial innervation of the cymba conchae by the auricular branch of the vagus nerve, providing an anatomical rationale for targeting this region with auricular neuromodulation (5, 6). However, conventional manual or seed-pressing auricular therapies are limited by operator-dependent variability and a lack of quantifiable stimulation parameters, which may reduce reproducibility and hinder their integration into standardized clinical practice (7).

To address these methodological limitations, transcutaneous auricular vagus nerve stimulation (taVNS) has emerged as a promising non-invasive neuromodulatory technique targeting auricular regions innervated by the auricular branch of the vagus nerve. The auricular concha, a principal target for taVNS because of its vagal afferent innervation, overlaps with regions traditionally used in TCM-based interventions for sleep regulation (8). By combining these auricular targets with modern neuromodulation technology, taVNS enables the application of quantifiable electrical parameters, including stimulation frequency, pulse width, stimulation intensity, and treatment duration. Compared with conventional manual or seed-pressing auricular therapies, taVNS therefore offers a more standardized, quantifiable, and reproducible approach to auricular stimulation (9).

Recent clinical trials and reviews suggest that taVNS may improve sleep quality and reduce insomnia severity in patients with chronic or primary insomnia, with a generally favorable safety profile (10, 11). Furthermore, functional neuroimaging studies indicate that taVNS may modulate brain regions and neural networks involved in hyperarousal, emotional processing, cognitive control, and autonomic regulation (12–14). Despite these encouraging findings, the existing evidence base remains limited, and the central neural mechanisms underlying the therapeutic effects of taVNS in CID have not been fully elucidated. In particular, longitudinal studies integrating clinical outcomes with neuroimaging measures remain scarce, limiting the concurrent evaluation of therapeutic effects and treatment-related neurobiological changes.

Resting-state functional magnetic resonance imaging (rs-fMRI) non-invasively characterizes spontaneous brain activity and intrinsic functional network organization by detecting blood oxygen level-dependent (BOLD) signal fluctuations. Accumulating evidence has revealed alterations in regional spontaneous neural activity and functional connectivity in patients with insomnia disorder. These abnormalities involve multiple brain regions and large-scale neural networks implicated in hyperarousal, emotion regulation, cognitive control, and self-referential processing, including the salience network, executive control network, and default mode network (15–17). Among established rs-fMRI metrics, the amplitude of low-frequency fluctuation (ALFF) and fractional amplitude of low-frequency fluctuation (fALFF) are widely used to characterize regional spontaneous brain activity (18). Alterations in ALFF/fALFF and functional connectivity have been reported in patients with CID (19– 20), supporting their utility for investigating CID-related brain dysfunction. Moreover, preliminary longitudinal rs-fMRI findings suggest that therapeutic interventions may be accompanied by changes in these functional measures (21), supporting their potential utility for investigating treatment-related neurobiological changes.

Accordingly, this study will **e**valuate the clinical efficacy and safety of taVNS in patients with CID and will incorporate prespecified exploratory rs-fMRI analyses to characterize brain functional alterations and treatment-related neuroimaging changes. Specifically, ALFF and fALFF will be used to examine regional spontaneous brain activity, including baseline differences between patients with CID and healthy controls and treatment-related changes in patients receiving active or sham-taVNS. Exploratory associations between neuroimaging changes and clinical outcomes will also be examined. By integrating clinical outcomes with neuroimaging measures, this protocol aims to more comprehensively characterize brain functional alterations in CID and to explore potential central mechanisms associated with taVNS treatment response.

## Study design and methods

### Study design

This is a single-center, randomized, sham-controlled clinical trial with an additional healthy control (HC) group. Eligible patients with chronic insomnia disorder (CID) will be randomized in a 1:1 ratio to either the active taVNS or the sham-taVNS group. Age- and sex-matched HCs will be recruited for baseline clinical and neuroimaging comparisons only and will not receive any intervention. Written informed consent will be obtained from all participants before any study-related procedures are performed.

The study protocol has been developed in accordance with SPIRIT guidelines, including the SPIRIT extension for Traditional Chinese Medicine. For patients with CID, the 16-week study period will consist of a 4-week intervention phase followed by a 12-week follow-up phase. Clinical assessments will be conducted at baseline and at weeks 2, 4, 8, 12, and 16. Resting-state functional magnetic resonance imaging (rs-fMRI) will be performed at baseline and Week 4, with the Week-4 scan conducted within 24 hours after the final intervention session. The overall study flowchart is presented in **Figure 1**, and the schedule of enrollment, interventions, and assessments is provided in **Table 1**.

**Figure 1:**
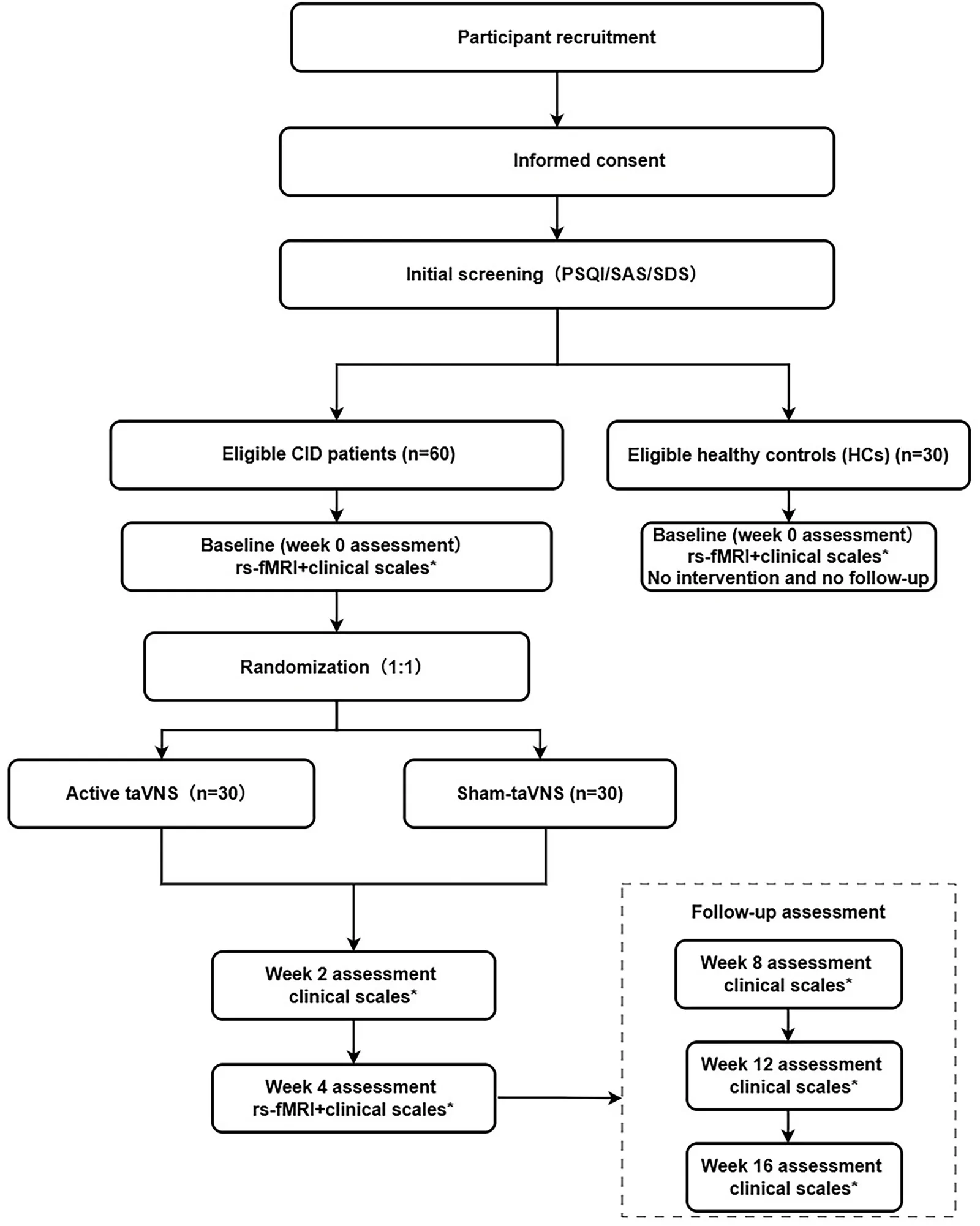
Flowchart of study process. *Clinical scales:* PSQI, Pittsburgh Sleep Quality Index; ISI, Insomnia Severity Index; ESS, Epworth Sleepiness Scale; SAS, Self-Rating Anxiety Scale; SDS, Self-Rating Depression Scale; HAMA, Hamilton Anxiety Rating Scale; HAMD, Hamilton Depression Rating Scale; MMSE, Mini-Mental State Examination; SF-36, 36-Item Short Form Health Survey; PSAS, Pre-Sleep Arousal Scale.

**Table 1:** Schedule of enrollment, interventions, and assessments.

Study period	Screening	Baseline	Treatment period		Follow-up		
Timepoint (Week)	-1	0	2	4	8	12	16
Enrollment							
Informed consent	✓						
Eligibility screening	✓						
Medical & demographic data	✓						
Randomization (CID patients only)		✓					
Interventions							
Active / Sham taVNS		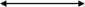					
Assessments							
rs-fMRI		✓		✓			
Clinical scales†		✓	✓	✓	✓	✓	✓
Sleep diary & adherence		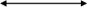					
Safety							
Safety evaluation / AE monitoring‡		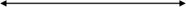					
Study completion							✓
Early termination		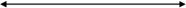					

† Clinical scales: PSQI, Pittsburgh Sleep Quality Index; ISI, Insomnia Severity Index; ESS, Epworth Sleepiness Scale; SAS, Self-Rating Anxiety Scale; SDS, Self-Rating Depression Scale; HAMA, Hamilton Anxiety Rating Scale; HAMD, Hamilton Depression Rating Scale; MMSE, Mini-Mental State Examination; SF-36, 36-Item Short Form Health Survey; PSAS, Pre-Sleep Arousal Scale.

‡ Adverse events (AEs) will be monitored and recorded at every visit and as they occur throughout the study period. Any serious adverse event (SAE) will be reported immediately to the Principal Investigator and the Institutional Review Board / Ethics Committee within 24 hours.

### Participants

Sixty eligible patients with chronic insomnia disorder (CID), with the diagnosis confirmed by at least two qualified specialists, are being recruited from the Departments of Sleep Medicine and Neurology at the Affiliated Traditional Chinese Medicine Hospital of Guangzhou Medical University. The planned recruitment period is from June 2025 to December 2026, and recruitment is currently ongoing. Thirty age- and sex-matched HCs will also be recruited for baseline clinical and neuroimaging assessments only.

### Diagnostic criteria

Patients with CID will be diagnosed with chronic insomnia disorder (CID) according to the International Classification of Sleep Disorders, 3rd edition (ICSD-3) criteria.

### Inclusion criteria

Eligible participants must meet all of the following criteria:

Patients with chronic insomnia disorder (CID).

- Diagnosis of CID according to ICSD-3 criteria.
- Right-handed, aged 18–70 years (inclusive).
- Pittsburgh Sleep Quality Index (PSQI) score > 7.
- Self-Rating Anxiety Scale (SAS) standard score < 70.
- Self-Rating Depression Scale (SDS) standard score < 73.

Able and willing to comply with study procedures and provide written informed consent.

Healthy controls (HCs).

- Right-handed, aged 18 to 70 years (inclusive).
- No history of mental or neurological disorders, as confirmed by the Mental Health Screening Scale and Neurosis Screening Scale.
- No current insomnia symptoms, as assessed using sleep scales before enrollment.

Able and willing to comply with study procedures and provide written informed consent.

### Exclusion criteria

Participants meeting any of the following criteria will be excluded:

Patients with chronic insomnia disorder (CID).

- Organic brain lesions, history of cranial surgery, or severe head trauma.
- Comorbid severe psychiatric disorders or serious systemic diseases (e.g., severe cardiovascular, hepatic, or renal disease).
- Use of anticholinergic drugs, non-steroidal anti-inflammatory drugs (NSAIDs), hormones, antipsychotics, or other central nervous system (CNS)-active medications within the 3 months before enrollment.
- History of alcohol or drug abuse.
- Contraindications to magnetic resonance imaging (MRI), such as an implanted cardiac pacemaker or insulin pump.
- Allergy to skin disinfectants or presence of skin damage/inflammation in the auricular region.
- Pregnant or lactating women.
- Participation in another clinical trial within the past 6 months.

Healthy controls (HCs).

- Contraindications to MRI scanning.
- History of alcohol or drug abuse.
- Allergy to skin disinfectants or presence of skin damage/inflammation in the auricular region.
- Pregnant or lactating women.
- Participation in another clinical trial within the past 6 months.

### Treatment adherence and prohibited concomitant treatments

For randomized patients with CID, poor adherence to study procedures, including failure to complete required assessments or adhere to the assigned intervention, as well as the use of prohibited concomitant treatments or interventions, will be documented as protocol deviations. Such deviations will not automatically result in withdrawal from the trial. Whenever possible, participants will continue to complete scheduled follow-up assessments and will be retained in the intention-to-treat (ITT) analysis in accordance with the prespecified statistical analysis plan. Participants with prespecified major protocol deviations may be excluded from the per-protocol (PP) analysis.

### Intervention discontinuation criteria

For randomized patients with CID, the assigned intervention may be discontinued for safety reasons if a participant develops a severe illness or experiences an intolerable adverse reaction that renders continued treatment clinically inappropriate. The Principal Investigator will evaluate such cases in accordance with the prespecified safety-monitoring procedures. Discontinuation of the assigned intervention will not automatically constitute withdrawal from the trial. Whenever feasible, participants who discontinue the intervention will continue to complete scheduled follow-up assessments and will remain in the ITT analysis.

### Participant withdrawal

All participants may voluntarily withdraw from the trial at any time and for any reason. For randomized patients with CID, participants who choose to discontinue the assigned intervention but agree to continued follow-up will be encouraged to complete the remaining scheduled assessments. If a participant withdraws consent for further study participation, no additional study procedures or follow-up assessments will be performed.

### Randomization and blinding

Eligible patients with chronic insomnia disorder (CID) will be randomized in a 1:1 ratio to either the active taVNS group or the sham-taVNS group using block randomization. The random allocation sequence will be generated by an independent investigator using SPSS version 24.0, with the block size(s) prespecified and concealed from personnel involved in participant recruitment, enrollment, and outcome assessment. The independent investigator will not be involved in participant recruitment, intervention delivery, outcome assessment, or data analysis. Allocation assignments will be placed in sequentially numbered, opaque, sealed envelopes and opened only after eligibility has been confirmed and baseline assessments have been completed, thereby maintaining allocation concealment until assignment.

Staff Member A will open the envelopes sequentially according to the order of participant enrollment and implement the assigned allocation. Staff Member A will be the only study staff member with access to treatment allocation and will be responsible for providing the standardized intervention training. Staff Member A will have no involvement in outcome assessment or data analysis.

Participants and outcome assessors will remain blinded to treatment allocation throughout the intervention and outcome-assessment periods. Staff Members B and C, who will conduct the clinical outcome assessments, will remain blinded to group allocation. To minimize the risk of inadvertent unblinding, intervention training and outcome assessments will be conducted by different study personnel in separate consultation rooms, and participants will be instructed not to disclose details of their stimulation sites to the outcome assessors. The active and sham-taVNS devices will be visually identical and will be operated using the same standardized procedures and stimulation parameters, with the stimulation location (*Liver/Kidney* auricular acupoints within the cymba conchae for active taVNS versus *Wrist/Elbow* auricular acupoints on the helix for sham stimulation) constituting the principal procedural difference between groups. Data analysts will receive group-coded datasets and remain blinded to treatment allocation until the prespecified primary analyses have been completed.

Following completion of the 4-week intervention, participants and outcome assessors will be asked to guess the treatment allocation as “active taVNS,” “sham-taVNS,” or “do not know.” Participant blinding will be quantitatively assessed using the Bang Blinding Index (BI) according to the prespecified statistical analysis plan, whereas outcome assessors’ allocation guesses will be summarized descriptively.

### Interventions

For patients with CID randomized to either intervention group, the first of the 56 prescribed stimulation sessions will be conducted at the hospital under the supervision of a trained physician. Before initiating home-based treatment, participants will receive standardized face-to-face training on the identification of their assigned auricular stimulation sites (**Figure 2**), correct placement of the ear-clip electrodes, operation of the stimulation device, adjustment of stimulation intensity, and relevant safety precautions. Participants will then self-administer the first stimulation session under physician supervision to confirm correct electrode placement and device operation. After demonstrating competency in performing the procedure, participants will take the stimulation device home and self-administer the remaining 55 sessions according to the assigned treatment protocol. An instructional video demonstrating the standardized operating procedure will also be provided as a reference throughout the 4-week home-based intervention period.

**Figure 2:**
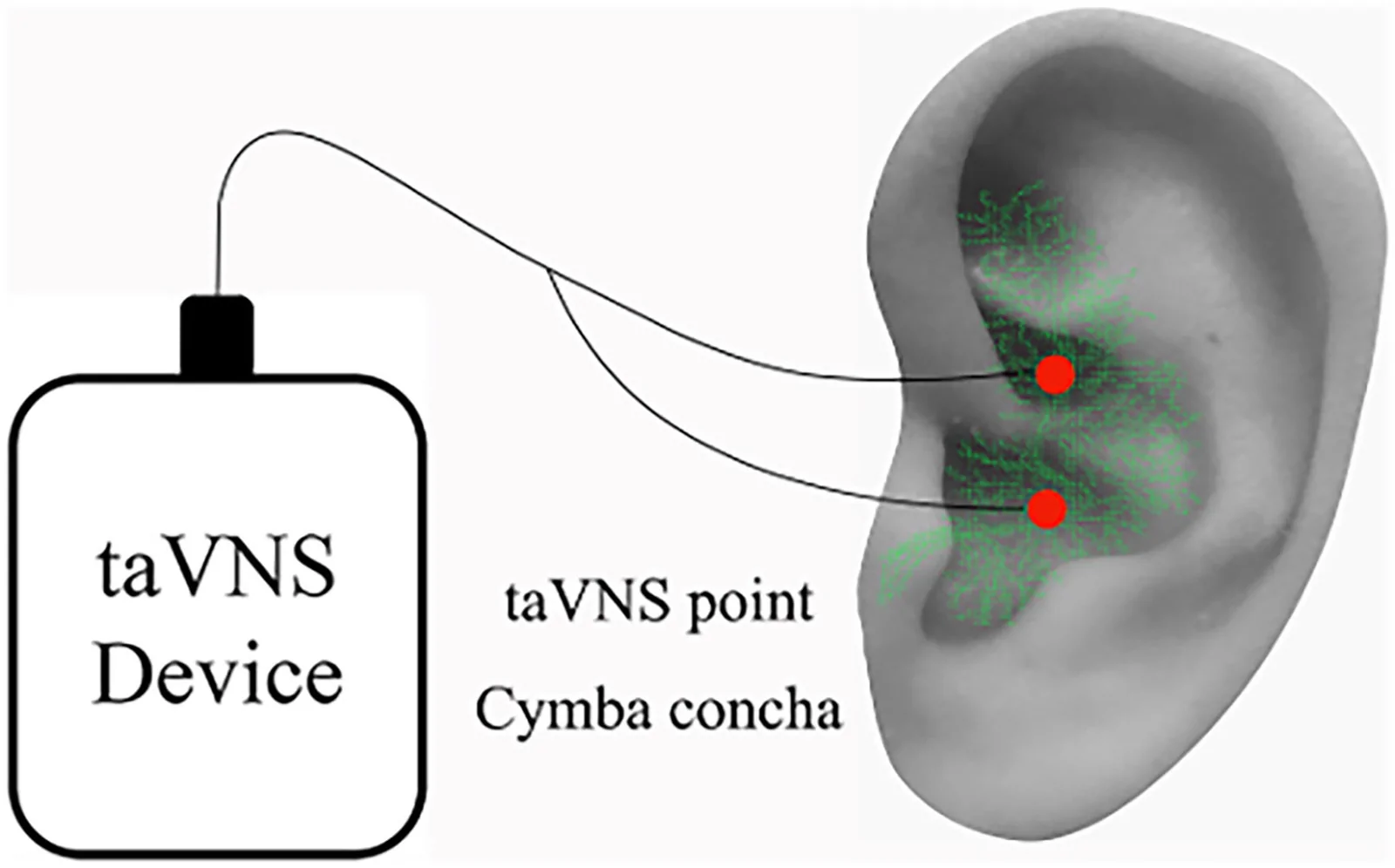
taVNS stimulation site on the left ear. Red dots indicate regions of taVNS (ABVN, green color). ABVN, auricular branch of the vagus nerve; taVNS, transcutaneous auricular vagus nerve stimulation.

### Active taVNS group

1. Acupoint Selection: The *Liver* and *Kidney* auricular acupoints within the cymba conchae will be selected bilaterally (**Figure 3**). The Liver acupoint is located at the posterior-inferior part of the cymba conchae. The *Kidney* acupoint is located in the cymba conchae, below the bifurcation of the superior and inferior crura of the antihelix.
2. Procedure: Prior to each session, the targeted acupoints and the metal contact surfaces of the ear-clip electrodes will be disinfected with 75% alcohol. The ear-clip electrodes will then be positioned bilaterally, with the metal contact surfaces placed directly over the selected auricular acupoints. The positive electrodes will be positioned over the bilateral Liver acupoints, and the negative electrodes will be positioned over the bilateral Kidney acupoints.
3. Stimulation parameters and intensity adjustment: Stimulation will be delivered using a dense-disperse waveform alternating between 20 Hz (disperse wave) and 100 Hz (dense wave), with a pulse width of 0.2 ms and an output voltage of 5.0 V. Stimulation intensity will be individually adjusted according to participant tolerance to a clearly perceptible but non-painful level below the individual pain threshold. During the initial supervised session, participants will be instructed by the physician on how to adjust the stimulation intensity appropriately. During subsequent home-based sessions, participants will follow the same procedure and will be instructed to reduce the stimulation intensity if pain or marked discomfort occurs. The prescribed waveform, frequency, pulse width, and output voltage will be standardized across participants, while stimulation intensity will be individualized according to tolerance. Participants will be instructed to follow the standardized operating procedure throughout the home-based intervention to promote treatment fidelity and reproducibility.
Frequency and Duration: Participants will receive two 30-minute sessions per day, one in the morning and one approximately 30 minutes before bedtime, 7 days per week for 4 weeks, totaling 56 sessions.

**Figure 3:**
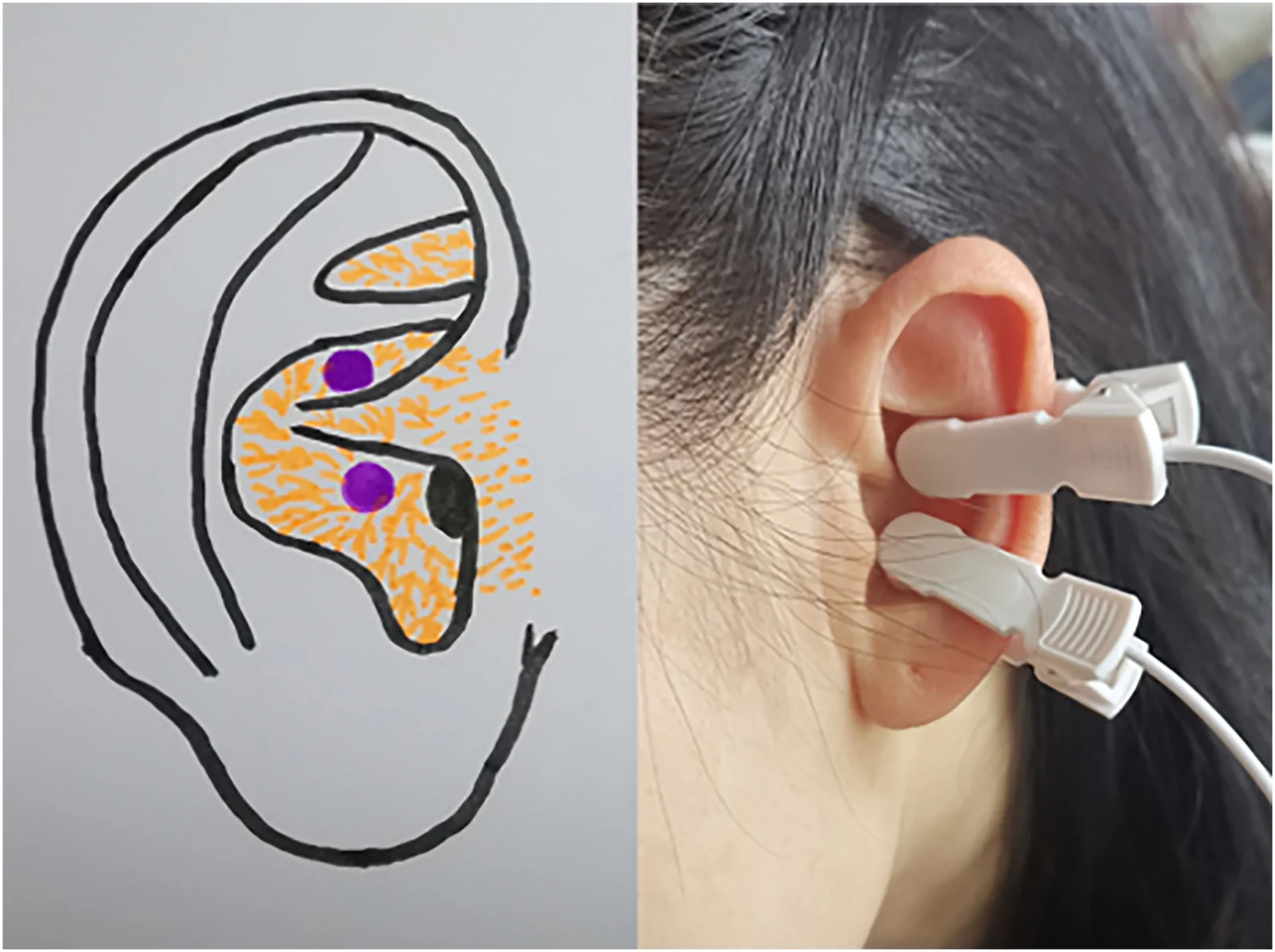
Site of transcutaneous auricular vagus nerve stimulation stimulation the auricular surface.

### Rationale and reproducibility of stimulation parameters

Stimulation parameters vary considerably across taVNS studies, including frequency, waveform, pulse width, intensity, stimulation site, and treatment schedule (**Table 2**). Previous insomnia studies have used 20 Hz or alternating 4/20 Hz stimulation with a pulse width of approximately 0.2 ms, and a 20/100-Hz dense-disperse waveform with the same pulse width has also been reported in taVNS neuroimaging research (6, 10–12, 22). Because no optimal stimulation regimen has yet been established, the present 20/100-Hz protocol is regarded as a prespecified, literature-supported approach rather than an established optimal regimen. All key stimulation parameters and treatment procedures will be standardized and documented to enhance reproducibility.

**Table 2:** Stimulation parameters across taVNS studies.

Study	Population	Stimulation site	Frequency / waveform	Pulse width	Stimulation intensity / adjustment	Treatment schedule
Jiao et al., 2020 (11)	Insomnia	Bilateral auricular concha	Alternating 4/20 Hz*	NR	Participant-adjusted	30 min, 5 days/week for 4 weeks
Wu et al., 2022 (10)	Primary insomnia	Bilateral auricular concha	20 Hz	0.2 ms	Individually adjusted according to tolerance	20 min, twice daily for a month
He et al., 2022 (12)	Chronic insomnia	Bilateral auricular concha	4/20 Hz	0.2 ms ± 30%	Adjusted according to subjective sensation	30 min, twice daily for 4 weeks
Zhang et al., 2024 (6)	CID	Bilateral cymba/cavum conchae	Alternating 4/20 Hz	0.2 ms ± 30%	0.8–1.5 mA; tolerance-based	30 min, twice daily 5 days/week, for 8 weeks
Fu et al., 2024 (22)	Parkinson's disease	Left cymba concha	20/100 Hz	0.2 ms	Tolerance-based	8 min during fMRI
Current study	CID	Bilateral cymba/cavum conchae	20/100 Hz dense disperse	0.2 ms	5.0-V output; individually adjusted below the pain threshold	30 min, twice daily for 4 weeks

CID, chronic insomnia disorder; fMRI, functional magnetic resonance imaging; NR, not reported; taVNS, transcutaneous auricular vagus nerve stimulation.

* Stimulation frequency alternated between 20 Hz for 10 s and 4 Hz for 5 s.

### Sham-taVNS group

Participants in the sham-taVNS group will receive bilateral transcutaneous electrical stimulation at the prespecified Wrist and Elbow auricular acupoints, selected to minimize direct stimulation of the principal auricular vagal target region while providing a sensory and procedural experience comparable to that of active taVNS (5, 11). The sham intervention is intended to control for nonspecific effects related to electrical stimulation, treatment procedures, and participant expectations rather than to function as a physiologically inert control. Accordingly, it will be regarded as an active sham control that may itself produce nonspecific sensory or physiological effects (5, 9).

1. Acupoint Selection: The *Wrist* and *Elbow* auricular acupoints on the helix will be selected bilaterally. Anatomically, these points are located by drawing an imaginary line along the scapha, connecting its superior tip to the level of the supratragic notch. The *Wrist* and *Elbow* acupoints are located at the second and third segments from the top, respectively.
2. Procedure and Parameters: Prior to each session, the targeted acupoints and the metal contact surfaces of the ear-clip electrodes will be disinfected with 75% alcohol. The ear-clip electrodes will be positioned bilaterally, with the metal contact surfaces placed directly over the selected sham auricular acupoints. The positive electrodes will be positioned over the bilateral Wrist acupoints, and the negative electrodes will be positioned over the bilateral Elbow acupoints. The same stimulation device will deliver the same prescribed electrical parameters as in the active taVNS group: a dense-disperse waveform alternating between 20 and 100 Hz, a pulse width of 0.2 ms, and an output voltage of 5.0 V. Stimulation intensity will be individually adjusted according to participant tolerance to a clearly perceptible but non-painful level below the individual pain threshold. Participants will receive the same standardized instruction regarding intensity adjustment as participants in the active taVNS group and will reduce the stimulation intensity if pain or marked discomfort occurs during home-based treatment. All other aspects of treatment delivery, including the initial supervised session, home-based self-administration, instructional video, and treatment schedule, will follow the same procedures as those used in the active taVNS group.
3. Frequency and Duration: Participants will receive two 30-minute sessions per day, one in the morning and one approximately 30 minutes before bedtime, 7 days per week for 4 weeks, totaling 56 sessions.

### Healthy control group

Healthy controls will not receive active or sham-taVNS and will undergo baseline clinical assessment and MRI acquisition only. Their baseline data will serve as reference data for cross-sectional comparisons with patients with CID.

### Adherence monitoring

During the 4-week home-based intervention, participants will receive daily treatment reminders and will document completion of the prescribed stimulation sessions through daily feedback and photographic check-ins. These records will be reviewed by the research team to monitor treatment adherence. A daily sleep diary will also be completed throughout the intervention and collected at the end of the treatment period. Adherence will be evaluated based on the documented completion of the prescribed stimulation sessions. The stimulation device does not provide retrievable electronic treatment logs; therefore, device-recorded session counts will not be available. Poor adherence will be documented as a protocol deviation but will not automatically result in withdrawal from the trial or exclusion from the intention-to-treat analysis, as described above.

### Observational indicators and follow-up

Demographic and clinical information will be collected at baseline. Patients with CID will undergo clinical assessments at baseline (Week 0), Weeks 2 and 4 during the 4-week intervention period, and Weeks 8, 12, and 16 during the subsequent 12-week follow-up period. Follow-up assessments will be completed online where applicable. Healthy controls will undergo baseline assessment only. The assessment schedule and outcome measures are summarized in **Table 1**.

### Primary outcome

The primary outcome is the change from baseline to week 4 in the Pittsburgh Sleep Quality Index (PSQI) score.

### Secondary outcomes

#### Clinical Outcomes

The prespecified secondary clinical outcomes will include the Insomnia Severity Index (ISI), Epworth Sleepiness Scale (ESS), Self-Rating Anxiety Scale (SAS), Self-Rating Depression Scale (SDS), Hamilton Anxiety Rating Scale (HAMA), Hamilton Depression Rating Scale (HAMD), Mini-Mental State Examination (MMSE), 36-Item Short Form Health Survey (SF-36), and Pre-Sleep Arousal Scale (PSAS). Changes from baseline to Week 4 will constitute the prespecified secondary clinical analyses, with multiplicity across these nine secondary outcomes controlled using the Holm correction. Assessments at Weeks 8, 12, and 16 will be used to evaluate the durability of clinical effects and will be treated as exploratory follow-up analyses.

#### Neuroimaging outcomes

Resting-state fMRI will be performed at baseline (Week 0) and after the 4-week intervention in patients with CID, with the Week-4 scan conducted within 24 hours after the final stimulation session, whereas healthy controls will undergo resting-state fMRI at baseline only. Because the study sample size was determined based on the primary clinical outcome (PSQI), all neuroimaging analyses will be considered exploratory. Both fractional amplitude of low-frequency fluctuation (fALFF) and amplitude of low-frequency fluctuation (ALFF) will be evaluated as prespecified exploratory neuroimaging measures. These measures will be used to characterize regional spontaneous brain activity associated with CID and to explore brain functional changes associated with active taVNS relative to sham-taVNS.

The prespecified exploratory neuroimaging analyses will include:

1. Baseline differences in ALFF/fALFF between patients with CID and healthy controls
2. Between-group differences in baseline-to-Week-4 changes in ALFF/fALFF between the active taVNS and sham-taVNS groups.
3. Exploratory associations between changes in ALFF/fALFF and changes in clinical outcomes.

### Feasibility outcomes

Operational feasibility will be assessed throughout the trial using prespecified indicators of recruitment yield, retention, treatment adherence, neuroimaging data usability, and tolerability. These indicators are intended to monitor trial conduct and identify potential implementation issues within the current trial and are not intended to serve as formal progression criteria for a future definitive RCT, The prespecified thresholds described below are operational feasibility benchmarks and, by themselves, will not determine participant inclusion in the intention-to-treat (ITT) or per-protocol (PP) analysis populations.

- Recruitment yield: Recruitment yield will be calculated as the number of eligible patients with CID who are enrolled and randomized divided by the total number of eligible patients with CID invited to participate, expressed as a percentage. A recruitment yield of ≥80% will be considered satisfactory; 50%–79% will trigger a review of recruitment barriers and relevant operational procedures; and <50% will indicate a major recruitment concern requiring formal review.
- Retention rate: Retention rate will be calculated as the number of randomized patients who complete the Week-4 primary outcome assessment divided by the total number of randomized patients, expressed as a percentage. A retention rate of ≥80% will be considered satisfactory; 50%–79% will trigger a review of reasons for withdrawal and consideration of participant-retention measures, such as improved visit scheduling, reminders, and follow-up coordination; and <50% will indicate a major retention concern requiring formal review. Retention at Weeks 8, 12, and 16 will also be descriptively reported to characterize participant retention during the follow-up period.λ
- Treatment adherence rate: Treatment adherence rate will be calculated as the number of documented completed stimulation sessions divided by the total number of prescribed stimulation sessions, expressed as a percentage. An adherence rate of ≥80% will be considered acceptable; 50%–79% will trigger a review of barriers to treatment adherence and consideration of measures such as scheduling optimization, participant reminders, and adherence support; and <50% will indicate a major adherence concern requiring formal review. These thresholds are intended for feasibility monitoring and will not, by themselves, constitute criteria for withdrawal from the trial or exclusion from the ITT analysis.
- Neuroimaging data usability: Neuroimaging data usability will be calculated as the number of acquired resting-state fMRI datasets meeting the prespecified quality-control criteria and usable for the planned analyses divided by the total number of acquired resting-state fMRI datasets, expressed as a percentage. A usability rate of ≥80% will be considered satisfactory; 50%–79% will trigger a review of the causes of unusable imaging data and reinforcement of standardized image-acquisition and quality-control procedures; and <50% will indicate a major concern regarding the neuroimaging component requiring formal review.
- Tolerability rate: Tolerability rate will be calculated as the number of participants who complete the 4-week intervention without permanent treatment discontinuation due to intervention-related intolerance divided by the number of participants who initiate the assigned intervention, expressed as a percentage. A tolerability rate of ≥80% will be considered acceptable; 50%–79% will trigger a safety and tolerability review and reinforcement of the prespecified individualized intensity-adjustment and adverse-event management procedures; and <50% will indicate a major tolerability concern requiring formal safety review.

For any feasibility metric falling within the 50%–79% range, the research team will conduct a structured review to identify the underlying causes and determine appropriate corrective actions. Such modifications will be limited primarily to operational aspects of trial conduct and will not alter treatment allocation, the primary outcome, core stimulation parameters, or the prespecified primary statistical analysis. Any proposed modification will require review and approval by the Principal Investigator before implementation. If a proposed modification constitutes a protocol amendment or affects information previously approved by the Ethics Committee, Ethics Committee approval will be obtained before implementation, as applicable. The trial registry will also be updated when changes affect registered study information, in accordance with applicable registry requirements. All modifications and their rationale will be documented.

A feasibility metric below 50% will trigger a formal review to determine whether further corrective measures, a protocol amendment, temporary suspension, or discontinuation of the relevant study component are warranted.

### Neuroimaging procedures

#### MRI acquisition and scanning procedure

Patients with CID will undergo MRI examinations at baseline (Week 0) and after the 4-week intervention (Week 4), whereas healthy controls will undergo MRI examination at baseline only. For patients with CID, the same imaging protocol and acquisition order will be used at both time points. Each examination will include a localizer scan, an 8-min resting-state BOLD fMRI scan, and a 4-min 37-s high-resolution three-dimensional (3D) T1-weighted BRAVO scan. The Week-4 MRI examination will be scheduled within 24 h after the final stimulation session.

MRI data will be acquired using a 3.0-T GE SIGNA Pioneer MRI scanner (GE Healthcare) equipped with a 21-channel Head/Neck coil.

Resting-state fMRI: Resting-state functional images will be acquired using an axial blood oxygen level-dependent (BOLD) echo-planar imaging sequence with the following parameters: repetition time (TR) = 2400 ms, echo time (TE) = 30 ms, flip angle = 90°, field of view (FOV) = 224 × 224 mm², acquisition matrix = 64 × 64, slice thickness = 3.5 mm, interslice gap = 0.5 mm, 40 axial slices, voxel size = 3.5 × 3.5 × 3.5 mm³, 200 volumes, and scan duration = 8 min. During resting-state fMRI acquisition, participants will be instructed to keep their eyes closed, remain awake and relaxed, avoid deliberate cognitive activity, and minimize head movement.
Structural MRI: High-resolution 3D T1-weighted structural images will be acquired using a BRAVO sequence in the sagittal plane with the following parameters: TR = 7.8 ms, TE = 3.2 ms, inversion time (TI) = 600 ms, flip angle = 12°, FOV = 256 × 256 mm², acquisition matrix = 256 × 256, slice thickness = 1.0 mm, 176 slices, voxel size = 1.0 × 1.0 × 1.0 mm³, number of excitations (NEX) = 1, and scan duration = 4 min 37 s.

### Resting-state fMRI preprocessing

Resting-state fMRI data will be preprocessed using SPM12 and DPARSF version 4.4. The first 10 volumes will be discarded to allow for signal equilibration and participant adaptation to the scanning environment. The remaining images will undergo slice-timing correction and realignment for head-motion correction. Individual fMRI datasets with head motion exceeding 2.5 mm of translation in any direction or 2.5° of rotation will be excluded from the corresponding neuroimaging analyses. The functional images will then be spatially normalized to Montreal Neurological Institute (MNI) space and smoothed using a 6-mm full-width at half-maximum (FWHM) Gaussian kernel. Linear detrending and nuisance-signal regression will subsequently be performed, with head-motion-related nuisance effects modeled using the Friston 24-parameter model.

ALFF and fALFF Calculation: Following preprocessing, the time series of each voxel will be transformed into the frequency domain using a fast Fourier transform (FFT) to obtain the power spectrum. The square root of the power spectrum will be calculated at each frequency, and ALFF will be derived from the mean amplitude across the 0.01–0.08 Hz frequency range. fALFF will be calculated as the ratio of the amplitude within the 0.01–0.08 Hz frequency range to the amplitude across the entire detectable frequency range.

For interindividual standardization, the ALFF and fALFF values of each voxel will be divided by the corresponding whole-brain mean ALFF and fALFF values, respectively, yielding mean-normalized ALFF (mALFF) and mean-normalized fALFF (mfALFF) maps for subsequent group-level statistical analyses. mfALFF will serve as the principal prespecified exploratory neuroimaging metric, whereas mALFF will be analyzed as a complementary exploratory measure.

### Neuroimaging statistical analysis

Group-level statistical analyses will be performed using the mALFF and mfALFF maps. Baseline differences between patients with CID and healthy controls will be assessed using whole-brain voxel-wise two-sample comparisons. Longitudinal between-group effects will be evaluated using the Group × Time interaction between the active taVNS and sham-taVNS groups across baseline and Week 4, testing whether baseline-to-Week-4 changes in mALFF and mfALFF differ between the two groups.

Exploratory brain–behavior analyses will examine associations between baseline-to-Week-4 changes in mALFF/mfALFF and corresponding changes in clinical outcomes in patients with CID. For the whole-brain analyses, statistical significance will be defined using an initial voxel-level threshold of P < 0.001, followed by cluster-level false discovery rate (FDR) correction at P < 0.05. As a sensitivity analysis, the same voxel-level threshold will be combined with cluster-level family-wise error (FWE) correction at P < 0.05. Multiple comparisons in the exploratory brain–behavior analyses will be controlled using the FDR method. Given that the study was not independently powered for neuroimaging outcomes, all neuroimaging findings will be interpreted as exploratory and hypothesis-generating.

### Safety monitoring and interim analysis

Given the relatively small sample size, single-center design, short intervention period, and non-invasive nature of taVNS, a formal Data Safety Monitoring Board (DSMB) will not be established, and no interim efficacy analysis is planned. Safety oversight will instead be provided by an independent safety monitor who is not involved in treatment delivery or outcome assessment. The independent safety monitor will review accumulated safety data, including AEs, SAEs, safety-related participant withdrawals, and the results of the Week-2 safety assessment. Any clinically important safety concern or unexpected pattern of intervention-related AEs will be promptly communicated to the Principal Investigator for evaluation and appropriate action in accordance with institutional procedures.

A formal safety assessment will be conducted at Week 2, in addition to the baseline and end-of-treatment (Week 4) assessments, to facilitate early identification of emerging safety concerns. Individual AEs will be managed according to the prespecified AE-management procedures, as detailed below.

### Safety outcomes and adverse event monitoring

Throughout the study, all adverse events (AEs) will be monitored and documented with respect to their onset, duration, severity, management, outcome, and assessed relationship to the study intervention. Based on a previous systematic review (23), commonly reported AEs associated with taVNS include ear pain, headache, dizziness, and local skin irritation or itching, whereas SAEs directly attributable to taVNS have rarely been reported. Given the twice-daily stimulation over 4 weeks and the clinical characteristics of insomnia, particular attention will also be paid to electrode-site contact dermatitis, changes in daytime somnolence, symptoms potentially related to excessive vagal stimulation (e.g., dizziness, nausea, bradycardia, and hypotension), and clinically relevant worsening or rebound of sleep symptoms after discontinuation of the intervention.

AE severity will be graded operationally as mild, moderate, or severe. Severity grading will be considered separately from the classification of an event as a serious adverse event (SAE). A mild AE is defined as a transient event causing minimal discomfort and no meaningful limitation of usual activities; treatment may be continued with close observation and symptomatic management as appropriate. A moderate AE is defined as an event causing some limitation of usual activities or requiring modification of the intervention; stimulation intensity may be reduced or treatment temporarily interrupted, with symptomatic management provided as appropriate. A severe AE is defined as an event causing marked functional impairment, requiring medical evaluation or intervention, or making continued stimulation clinically inappropriate; the study intervention will be discontinued, and appropriate medical evaluation and management will be provided. If symptoms suggestive of excessive vagal stimulation occur, the participant will undergo prompt clinical assessment and further evaluation as clinically indicated.

All AEs will be followed until resolution, stabilization, or an appropriate clinical endpoint, and their relationship to the study intervention will be assessed and documented. Safety will be formally assessed at baseline, Week 2, and Week 4, while AEs will be assessed and recorded throughout the intervention and follow-up periods, including the scheduled follow-up visits at Weeks 8, 12, and 16. Any SAE will be reported to the Principal Investigator and the Ethics Committee within 24 hours of awareness and managed in accordance with institutional procedures.

### Statistical considerations

#### Sample size

The sample size calculation was based on a two-group comparison of mean PSQI change scores. The target between-group difference in PSQI reduction was set at 3.0 points, representing a clinically meaningful difference based on prior literature (24). Based on a prior randomized controlled trial in patients with insomnia, the standard deviation was set at 3.35 (25). Assuming a two-sided α = 0.05 and 90% power, 27 participants per group were required. Accounting for a 10% dropout rate, the sample size was increased to 30 participants per group. Thus, 60 patients with chronic insomnia disorder (30 in the active taVNS group and 30 in the sham-taVNS group) will be randomized, and an additional 30 healthy controls will be enrolled as a non-randomized healthy control group for baseline reference comparisons. The sample size calculation applies to the randomized comparison between the active taVNS and sham-taVNS groups and was based on the primary clinical outcome (PSQI); the healthy controls are not included in this efficacy sample-size calculation. The prespecified neuroimaging analyses were not independently powered and will therefore be interpreted as exploratory and hypothesis-generating.

### Clinical data analysis

Clinical statistical analyses will be performed using IBM SPSS Statistics version 26.0 (IBM Corp., Armonk, NY, USA). All statistical tests will be two-sided, with P < 0.05 considered statistically significant unless otherwise specified.

- Baseline characteristics: Continuous variables will be summarized as mean ± standard deviation (SD) when approximately normally distributed and as median (P25–P75) otherwise. Baseline characteristics will be summarized descriptively for each randomized intervention group, and formal significance testing between the active taVNS and sham-taVNS groups will not be used to assess the success of randomization. For baseline cross-sectional comparisons involving healthy controls, normally distributed continuous variables will be compared using appropriate analysis of variance or two-sample methods, depending on the comparison of interest, with Bonferroni-adjusted *post hoc* comparisons when applicable. Non-normally distributed continuous variables will be compared using the Kruskal–Wallis test or the corresponding two-sample nonparametric test, as appropriate. Categorical variables will be presented as frequencies and percentages and compared using Pearson’s chi-square test or Fisher’s exact test, as appropriate.
- Primary outcome analysis: The primary efficacy analysis will compare the active taVNS and sham-taVNS groups with respect to PSQI scores at Week 4 using analysis of covariance (ANCOVA), with the Week-4 PSQI score as the dependent variable, treatment group as the fixed effect, and baseline PSQI score as a covariate. The adjusted between-group difference, corresponding 95% confidence interval(CI), and two-sided P value will be reported. Although the primary outcome is defined clinically as the change in PSQI score from baseline to Week 4, baseline-adjusted ANCOVA will be used for primary statistical inference because it accounts for baseline variation and generally provides greater statistical precision than an unadjusted comparison of change scores.
- Secondary clinical outcome analyses: The nine prespecified secondary clinical outcomes at Week 4 (ISI, ESS, SAS, SDS, HAMA, HAMD, MMSE, SF-36, and PSAS) will be analyzed using analogous baseline-adjusted ANCOVA models, with the corresponding Week-4 score as the dependent variable, treatment group as the fixed effect, and the corresponding baseline score as a covariate. Adjusted between-group differences with 95% CIs will be reported. Changes at Week 2 will be examined as supportive/exploratory analyses of early treatment response, whereas assessments at Weeks 8, 12, and 16 will be used for exploratory analyses of the durability of treatment effects.
- Longitudinal clinical outcome analyses: Longitudinal trajectories of clinical outcomes across the post-baseline assessments at Weeks 2, 4, 8, 12, and 16 will be examined, as applicable to the assessment schedule for each outcome, using linear mixed-effects models for repeated measures. The corresponding baseline score will be included as a covariate. Treatment group, time, and the treatment-group-by-time interaction will be included as fixed effects, and participant will be included as a random effect to account for within-participant correlation across repeated measurements. The treatment-group-by-time interaction will be used to evaluate whether longitudinal changes differ between the active taVNS and sham-taVNS groups. These longitudinal analyses will be considered supportive or exploratory, as appropriate, and will not alter the prespecified primary efficacy comparison at Week 4.
- Multiple comparisons: The single primary hypothesis will be tested at a two-sided α level of 0.05 without multiplicity adjustment. For the nine prespecified secondary clinical outcomes at Week 4 (ISI, ESS, SAS, SDS, HAMA, HAMD, MMSE, SF-36, and PSAS), multiplicity will be controlled as a single family using the Holm procedure at a family-wise two-sided α level of 0.05. Both nominal and multiplicity-adjusted P values will be reported. Analyses of these secondary clinical outcomes at time points other than Week 4 (Weeks 2, 8, 12, and 16) will be considered supportive or exploratory, as appropriate, and will be interpreted primarily on the basis of effect estimates and 95% CIs rather than statistical significance alone.
- Correlation analyses: Where correlation analyses are performed for clinical variables, Pearson’s or Spearman’s correlation coefficients will be used, as appropriate, based on the distributional characteristics of the data and the nature of the association being examined. These analyses will be regarded as exploratory unless otherwise prespecifiedλ.
- Missing data: The primary efficacy analysis will follow the intention-to-treat (ITT) principle and will include all randomized participants according to their originally assigned treatment groups. Missing primary-outcome data will be addressed using multiple imputation under a missing-at-random assumption, with complete-case analysis performed as a sensitivity analysis. For longitudinal mixed-effects analyses, all available repeated measurements will be included, with inference relying on the missing-at-random assumption underlying the model. Discontinuation of the assigned intervention, poor adherence, or other protocol deviations will not by themselves result in exclusion from the ITT analysis. A per-protocol (PP) analysis will be conducted as a sensitivity analysis using participants without prespecified major protocol deviations. The operational feasibility adherence thresholds described above will not, by themselves, define eligibility for the PP population.
- Blinding assessment: Participant blinding effectiveness will be evaluated using the Bang Blinding Index (BI) separately for each intervention group, with corresponding 95% CIs (6, 26, 27). A BI of 0 indicates guessing consistent with chance, whereas positive and negative values indicate tendencies toward correct and incorrect identification, respectively. A BI between −0.2 and 0.2 will be interpreted as being consistent with successful blinding (6, 27). Values outside this range will be considered indicative of potential departure from successful blinding and will be interpreted together with the direction of the BI and its 95% CI. If potential unblinding is detected, it will be transparently reported; the prespecified primary efficacy analysis will remain unchanged, and the potential influence of unblinding will be considered when interpreting the treatment effects. Outcome assessors’ allocation guesses will be summarized descriptively.

### Data management and quality control

- Staff will receive standardized training in the study protocol, Good Clinical Practice (GCP), device operation, and outcome assessment. Only personnel who have completed the required training will perform study-related procedures, with refresher training provided every 6 months.
- The prescribed waveform, stimulation frequency, pulse width, and device output will be standardized according to the assigned intervention protocol, and tolerance-based intensity adjustment will follow the prespecified procedures. Participants will receive standardized training before home-based treatment. Adherence will be monitored through daily treatment reminders, participant feedback, photographic check-ins, and documented completion of prescribed stimulation sessions. Device function will be checked at scheduled follow-up visits.
- Study data will be collected using standardized case report forms (CRFs), identified only by a unique study ID, and entered or completed within 24 h of the corresponding study visit or assessment whenever applicable.
- Data will undergo double entry into a secure electronic data capture (EDC) system with predefined range and logic checks. An independent monitor will verify a randomly selected 10% of CRFs against the corresponding source data, and identified discrepancies will be documented, queried, and resolved before database lock.
- Study data will be stored securely, with access restricted to authorized study personnel, including the Principal Investigator and designated data managers. The database will be locked after completion of data cleaning and resolution of outstanding queries.

### Ethics and dissemination

The study protocol has been approved by the Ethics Committee of the Affiliated Traditional Chinese Medicine Hospital of Guangzhou Medical University (Approval No. 2024NK56) and will be conducted in accordance with the Declaration of Helsinki, Good Clinical Practice, applicable Chinese regulations, and institutional requirements. Written informed consent will be obtained from all participants before enrollment and before any study-specific procedures are performed.

The study findings will be disseminated through peer-reviewed publications and scientific presentations. Trial registration information will be maintained and updated as required. Authorship will follow the recommendations of the International Committee of Medical Journal Editors (ICMJE), and no professional medical writers will be involved in manuscript preparation.

## Discussion

Sleep is a fundamental physiological process essential for physical health, cognitive function, and psychological well-being. Epidemiological evidence suggests that chronic insomnia disorder (CID) affects approximately 10% of adults and is associated with an increased burden of physical and psychological comorbidities (28). Current management strategies for CID include both pharmacological and non-pharmacological interventions. Although pharmacological treatments may provide short-term symptomatic relief, concerns regarding residual daytime effects, tolerance, dependence, and inappropriate long-term use may limit their sustained application (29). These limitations highlight the need for safe, accessible, and home-applicable non-pharmacological treatment strategies.

The vagus nerve is a major component of the autonomic nervous system and plays an important role in the regulation of homeostasis. Vagus nerve stimulation (VNS) has therefore attracted increasing interest as a potential neuromodulatory approach for sleep and related disorders. Conventional invasive VNS requires surgical implantation and may be associated with procedure- or stimulation-related adverse effects, including cough and hoarseness (30). In contrast, transcutaneous auricular vagus nerve stimulation (taVNS) provides a non-invasive approach by targeting auricular regions innervated by vagal afferent fibers, particularly the conchal region. Although its mechanisms in insomnia remain incompletely understood, emerging evidence suggests that taVNS may influence autonomic regulation, inflammatory processes, and brain regions or functional networks involved in arousal, emotion regulation, and sleep-related processing (21). In the present study, rs-fMRI-derived ALFF and fALFF measures will therefore be used to characterize regional spontaneous brain activity associated with CID and to explore brain functional changes associated with active taVNS relative to sham-taVNS.

From the perspective of Traditional Chinese Medicine (TCM), auricular therapy regards the ear as a microsystem reflecting the functional state of the body. Within this theoretical framework, the *Liver* is traditionally considered to regulate the free flow of *Qi* and emotional activity, whereas the *Kidney* is considered to store essence and nourish the brain. On this basis, the *Liver* and *Kidney* auricular acupoints were selected as the stimulation sites in the present protocol. These acupoints are located within the cymba conchae, an auricular region with vagal afferent innervation. Thus, the selected stimulation sites provide a conceptual intersection between the traditional rationale for auricular acupoint selection and the neuroanatomical basis of auricular vagal stimulation. The clinical and neuroimaging analyses in this trial may help explore whether stimulation at these sites is associated with improvements in insomnia symptoms and corresponding changes in spontaneous brain activity.

Several limitations of this protocol should be acknowledged. First, the 4-week intervention period may be insufficient to fully characterize durability of treatment effects, although clinical follow-up will continue through Week 16. Second, the single-center design and relatively modest sample size may limit the generalizability of the findings. The sample size was determined on the basis of the primary clinical outcome rather than the neuroimaging outcomes; consequently, the prespecified fMRI analyses may be underpowered to detect small-to-moderate effects and will be interpreted as exploratory and hypothesis-generating rather than confirmatory.

Third, the Week-4 MRI examination will be conducted within 24 h after the final stimulation session. Neuroimaging changes observed at this time point may therefore reflect a combination of recent stimulation-related effects and cumulative effects of the 4-week intervention, which cannot be fully distinguished within the present design. In addition, healthy controls undergo MRI at baseline only, precluding direct estimation of four-week test–retest changes in ALFF and fALFF in individuals without CID.

Fourth, although the Wrist and Elbow auricular acupoints used for sham-taVNS were selected outside the principal auricular vagal target region to reduce direct vagal stimulation while maintaining a comparable sensory and procedural experience, these sites are recognized auricular acupoints. The sham condition therefore represents an active sham rather than a physiologically inert control, and nonspecific somatosensory, expectancy-related, or acupoint-related effects cannot be completely excluded. This should be considered when interpreting between-group differences.
